# On‐surface Synthesis of a Chiral Graphene Nanoribbon with Mixed Edge Structure

**DOI:** 10.1002/asia.202001008

**Published:** 2020-10-12

**Authors:** Ashok Keerthi, Carlos Sánchez‐Sánchez, Okan Deniz, Pascal Ruffieux, Dieter Schollmeyer, Xinliang Feng, Akimitsu Narita, Roman Fasel, Klaus Müllen

**Affiliations:** ^1^ Department of Chemistry The University of Manchester Oxford road Manchester M13 9PL UK; ^2^ Empa Swiss Federal Laboratories for Materials Science and Technology 8600 Dübendorf Switzerland; ^3^ ESISNA Group, Materials Science Factory Institute of Materials Science of Madrid (ICMM–CSIC) Sor Juana Inés de la Cruz 3 28049 Madrid Spain; ^4^ Department of Chemistry Johannes Gutenberg-University 55099 Mainz Germany; ^5^ Center for Advancing Electronics Dresden (cfaed) & Faculty of Chemistry and Food Chemistry Technische Universität Dresden 01062 Dresden Germany; ^6^ Max Planck Institute for Polymer Research Ackermannweg 10 55128 Mainz Germany; ^7^ Department of Chemistry and Biochemistry University of Bern 3012 Bern Switzerland

**Keywords:** graphene nanoribbon, chiral edge, on-surface synthesis, polycyclic aromatic hydrocarbon, scanning tunneling microscopy and spectroscopy

## Abstract

Chiral graphene nanoribbons represent an important class of graphene nanomaterials with varying combinations of armchair and zigzag edges conferring them unique structure‐dependent electronic properties. Here, we describe the on‐surface synthesis of an unprecedented cove‐edge chiral GNR with a benzo‐fused backbone on a Au(111) surface using 2,6‐dibromo‐1,5‐diphenylnaphthalene as precursor. The initial precursor self‐assembly and the formation of the chiral GNRs upon annealing are revealed, along with a relatively small electronic bandgap of approximately 1.6 eV, by scanning tunnelling microscopy and spectroscopy.

## Graphical abstract

**Tailoring the edge structure of graphene nanoribbons**: Design and synthesis of chiral graphene nanoribbons with mixture of zig‐zag, armchair and cove type edges. This work expands the structural variety of GNRs, providing an insight into the non‐planarity induced by the cove edge geometry and the formation of chiral GNRs.

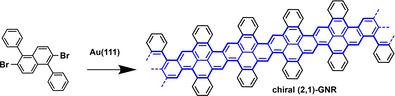

Graphene nanoribbons (GNRs), ribbon‐shaped cut‐outs of graphene with nanometre‐scale widths, have attracted much attention due to their unique (opto)electronic properties, and have been recognized as a promising material for next‐generation electronics.^[1]^ Their properties, such as chemical stability, optical absorption and emission, charge‐carrier mobility, and spin state, heavily depend on their geometrical features such as edge structure, width, and length.[^1e^, ^1f^, ^1j^, ^2^] The edge structure is the most critical factor for the properties of GNRs, making armchair‐edge GNRs (AGNRs) semiconducting with width‐dependent bandgaps and zigzag‐edge GNRs (ZGNRs) potentially magnetic thanks to their edge‐localized states that can be spin polarized.[^2a^, ^3^] Chiral GNRs (ch‐GNRs) combining armchair and zigzag edges are characterized by a chiral vector (*n*,*m*) which connects crystallographically equivalent sites along the edge and defines the edge structure of the GNR.^[4]^ These ch‐GNRs are theoretically predicted to present electronic band structures and magnetic properties dependent on the chiral vector.[^2d^, ^5^] In addition to armchair and zigzag edge structures, one can also consider other geometries like cove and fjord edges,^[6]^ which result in non‐planarity due to the repulsion between neighbouring hydrogen atoms.^[7]^ Because structural control at the single‐atom level is essential to obtain GNRs with desired properties, conventional top‐down nanofabrication methods (e. g., patterning of graphene or unzipping of carbon nanotubes) fail to furnish GNRs with defined and singly hydrogenated edges.[^2e^, ^8^] In contrast, bottom‐up synthesis can provide GNRs with atomically precise widths, edge structures and chemical functionalization, thus attracting increasing attention over the past decade.[^1a^, ^1f^, ^9^]

The bottom‐up synthesis can be carried out using tailor‐made molecular precursors, typically through two methods, namely i) in solution, by the methods of organic and polymer chemistry,[^1f^, ^1k^, ^2f^, ^10^] and ii) on surfaces, by using tools and strategies of surface science and on‐surface synthesis.[^1e^, ^1f^, ^1j^, ^2g^, ^2h^, ^2i^, ^11^] In particular, the on‐surface protocol allows for the in‐situ visualization of the resulting GNRs by high‐resolution scanning probe microscopies, while unambiguous structural elucidation of solution‐synthesized GNRs remains challenging.[^1a^, ^1e^, ^2h^, ^11^, ^12^] Through on‐surface synthesis, AGNRs with varying widths have been achieved, exhibiting distinct bandgaps in agreement with theoretical predictions.[^1e^, ^1i^, ^13^] Formation of a ZGNR with perfect zigzag edge structure was also unambiguously demonstrated, along with the presence of edge‐localized electronic states.^[11a]^ A cove‐edge GNR was prepared by using a dibromobichrysene precursor, demonstrating its non‐planar structure on a surface.^[7a]^ In regards to ch‐GNRs, chiral (3,1)‐GNRs were synthesized using bianthryl‐based precursors,^[14]^ and a heteroatom‐doped chiral (4,1)‐GNR, having oxygen‐boron‐oxygen segments on the zigzag edge, was also reported.^[15]^ However, there are still scarce examples of ch‐GNRs and cove‐edge GNRs reported to date. Here, we describe the on‐surface bottom‐up synthesis of unique ch‐GNR **9** that can be considered as a benzo‐fused chiral (2,1)‐GNR having mixed armchair and cove edges. The 2,6‐dibromo‐1,5‐diphenylnaphthalene (NAP **7**) molecule serves as the precursor to ch‐GNR **9** growth under ultrahigh vacuum (UHV) conditions on a Au(111) surface. Characterization by means of scanning tunneling microscopy and spectroscopy (STM and STS) shows the non‐planar structure of ch‐GNR **9** and reveals a bandgap of ∼1.6 eV.

For the synthesis of NAP **7**, 2,6‐dimethoxy naphthalene (**1**) was selectively brominated with *N*‐bromosuccinimide to give 1,5‐dibromo‐2,6‐dimethoxynaphthalene (**2**)^[16]^ in 98% yield, followed by a Suzuki coupling reaction with phenylboronic acid to produce 2,6‐dimethoxy‐1,5‐diphenylnaphthalene (**3**) in 90% yield (Scheme 1). Demethylation of **3** was achieved by treatment with boron tribromide (BBr_3_) to provide 2,6‐dihydroxy‐1,5‐diphenylnaphthalene (**4**) in 95% yield, which was subsequently reacted with trifluoromethanesulfonic anhydride to afford bistriflate **5** in 87% yield. Miyaura borylation of **5** gave bisboronic ester **6** in 61% yield along with mono‐substituted byproduct (∼30% yield). Treatment of **6** with CuBr_2_ in a mixture of diaoxane and methanol under reflux allowed to convert the boronic ester to the bromide, providing NAP **7** in 73% yield (see ESI for reaction conditions and characterization details).

**Scheme 1:**
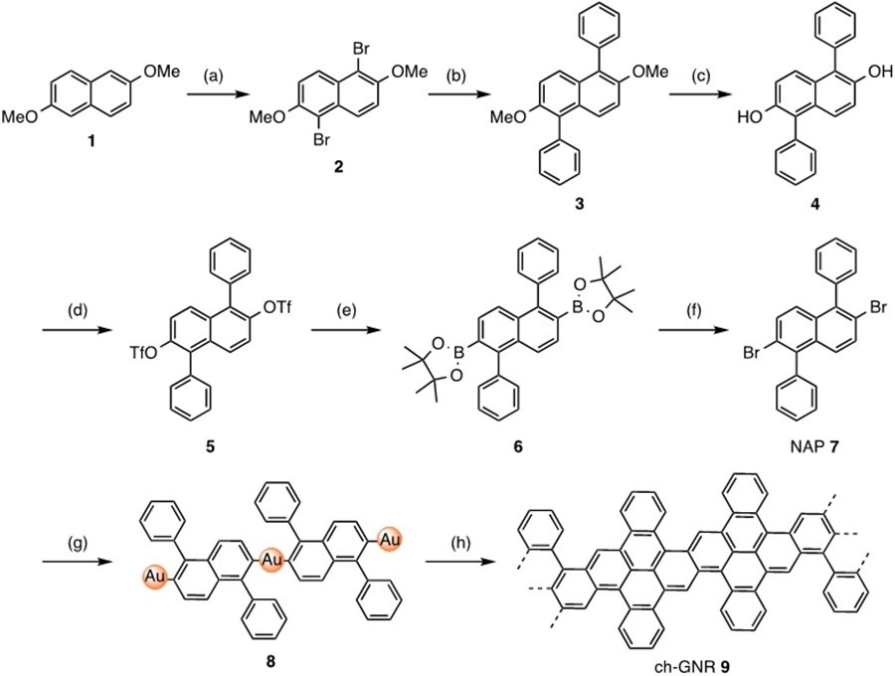
Synthesis of 2,6‐dibromo‐1,5‐diphenylnaphthalene (NAP **7**) as the precursor used in UHV‐deposition and subsequent on‐surface synthesis of ch‐GNR **9**. Reagents and conditions: (a) *N*‐bromosuccinamide, chloroform, room temperature, 10 h, 98%; (b) phenylboronic acid, K_2_CO_3_, toluene, Pd(PPh_3_)_4_, 90 °C, 12 h, 90%; (c) BBr_3_, dichloromethane, room temperature, 12 h, 95%; (d) trifluoromethanesulfonic anhydride, triethylamine, dichloromethane, 50 °C, 10 h, 87%; (e) Pinacolborane, Pd(dppf)Cl_2_, dioxane, reflux, 16 h, 61%; (f) CuBr_2_, dioxane/methanol/water, reflux, 24 h, 73%; (g) Au(111), UHV chamber, 470 K, 20 min; (f) Au(111), UHV chamber, 570 °C, 90 min.

Prior to the on‐surface synthesis, NAP **7** was recrystallized twice from a mixture of dichloromethane (DCM) and methanol to yield colourless needle‐shape crystals, which also allowed for the single‐crystal X‐ray analysis (Figure 1). The NAP molecules were packed in a P‐1 (triclinic) space group and the unit cell contained two independent molecules with *C*_i_ symmetry (Figure 1b and c). This dyad displayed partial π‐π interaction (3.4 Å) between the two naphthalene units. Hydrogen atoms at the *ortho*‐position of each phenyl ring were participating in C−H‐π (2.96 Å) interactions with the other naphthalene unit in the dyad. Intermolecular C−H⋅⋅⋅Br interactions (3.01 Å) were also observed between NAP molecules in neighbouring dyads.

**Figure 1:**
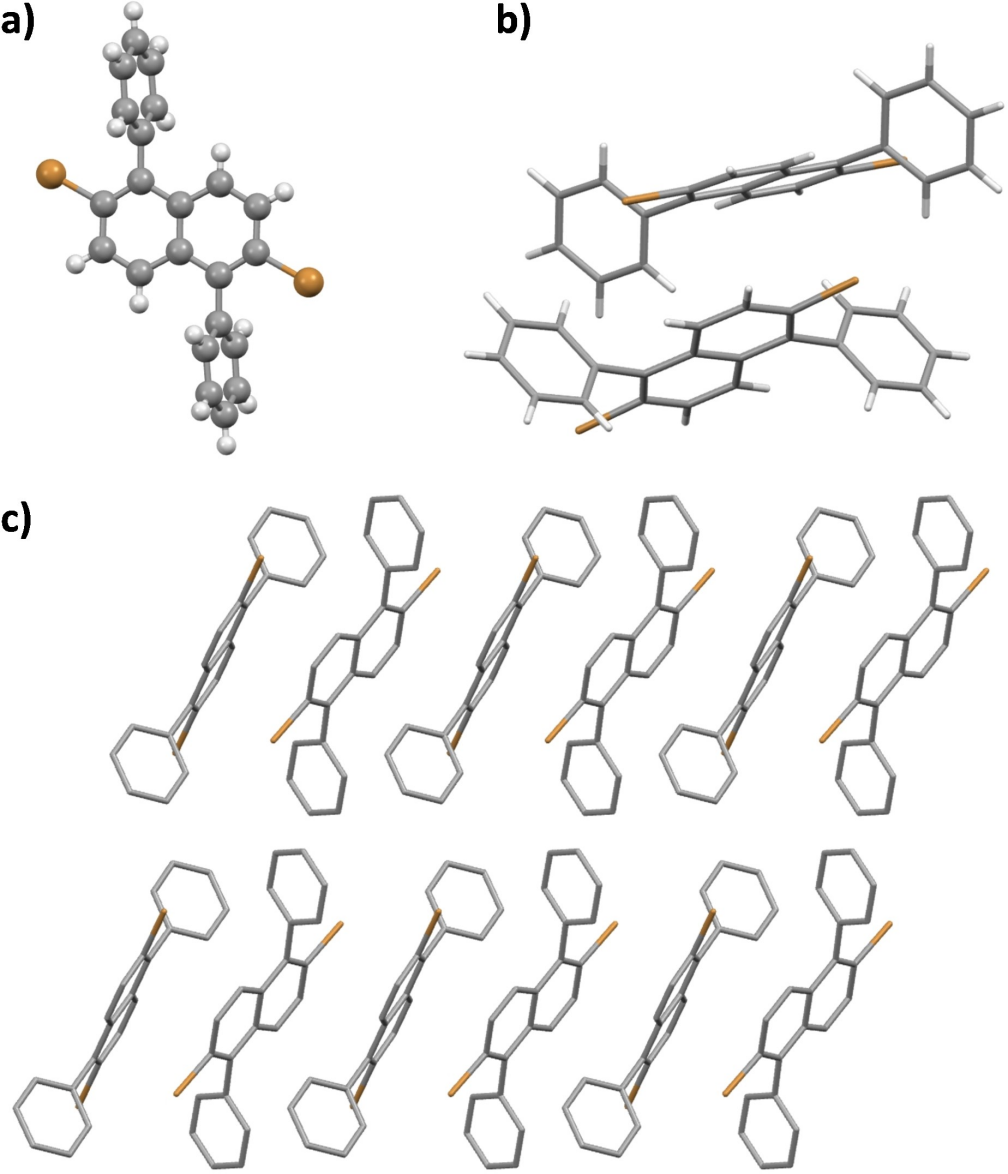
Single‐crystal X‐ray analysis of NAP **7**. a) Front view; b) Dyad of NAP; c) Crystal packing in crystallographic a‐axis.

For the on‐surface synthesis of **9**, NAP **7** was thermally deposited onto a Au(111) substrate held at room temperature under UHV conditions. As‐deposited NAP molecules self‐assembled into homochiral islands, presumably stabilized by intermolecular Br⋅⋅⋅H−C hydrogen bonds (Figure 2a and 2b).^[17]^ Subsequent annealing of the gold substrate at 470 K for 20 minutes led to the formation of bunches of long chains, as revealed by STM analysis (Figure 2c and d). The presence of faint lines on the herringbone reconstruction indicated the debromination of NAP molecules already at this temperature (see Figure S1 in ESI). However, measured intermolecular distances (1.1 nm) in these chains were larger than the value of 0.6 nm for the corresponding polymers expected after the aryl‐aryl coupling (see superimposed scaled chemical structures in Figure 2d), suggesting the formation of a metal‐coordinated intermediate **8**. Interestingly, several bright protrusions are observed in Figure 2d specifically where there is a change in the stacking symmetry along the supramolecular chains. Given the large distance between monomers in this phase, these protrusions are attributed to the presence of Br atoms underneath specific sites of the polymer, as reported for other intermediate states of GNRs,^[18]^ maybe related to stacking faults originated by the proximity of monomers of distinct surface chirality.

**Figure 2:**
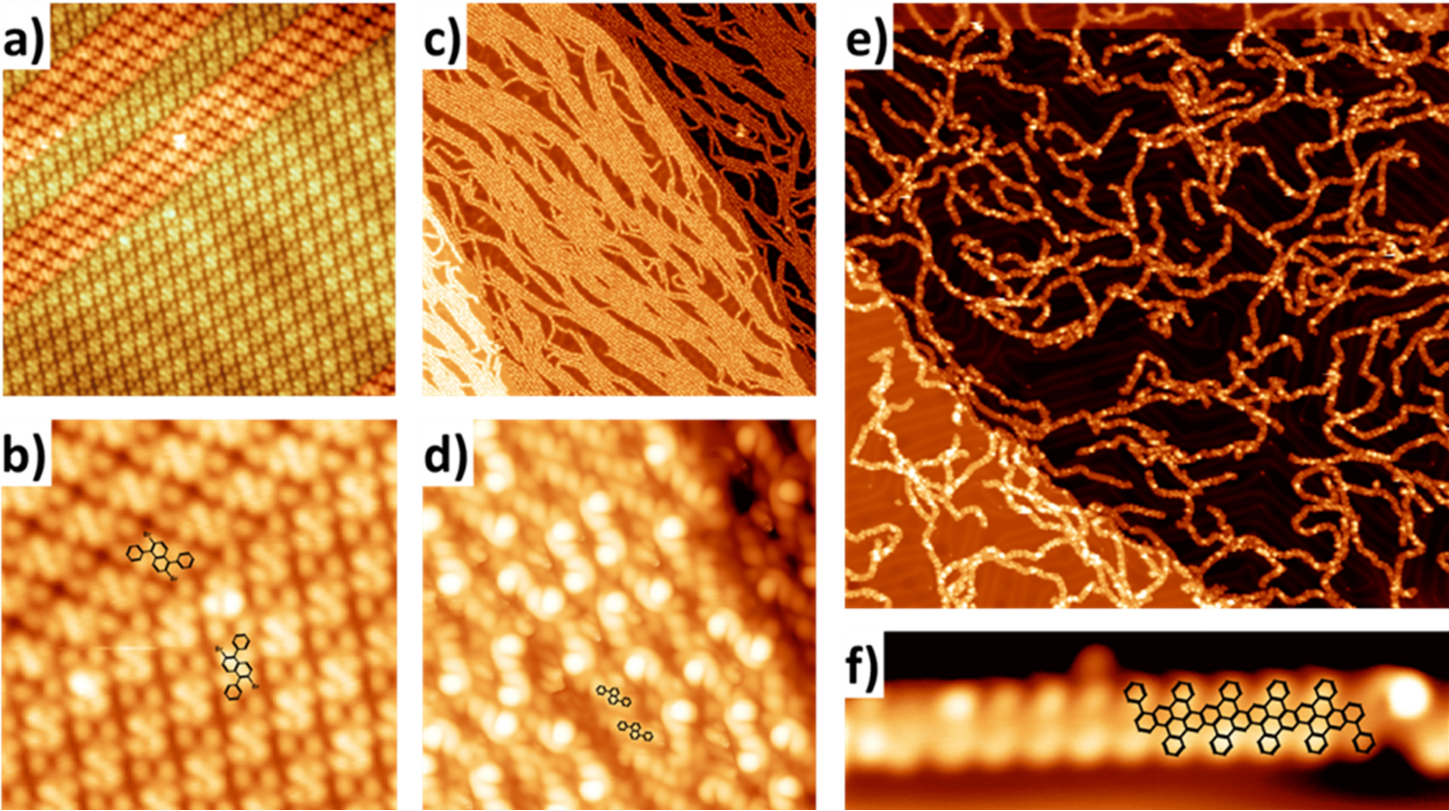
STM images monitoring the on‐surface reactions of NAP **7** to form ch‐GNR **9**: a) STM image of the as‐deposited NAP **7** molecules at room temperature. Greenish shadowing highlights molecules in second homochiral domain. STM parameters: (20 nm×20 nm) 0.03 nA, −1.5 V, 77 K. b) Zoom (6.5 nm×6.5 nm) of STM image in panel a) showing the molecular orientation in the two domains. c) STM image after annealing the sample at 470 K. STM parameters: (150 nm×150 nm) 0.04 nA, 1.0 V, 77 K. d) High‐resolution STM image of the chains in panel c), with scaled chemical structures superimposed. STM parameters: (12 nm×12 nm) 0.04 nA, −1.2 V, 77 K. e) STM image after annealing at 570 K. STM parameters: (140 nm×140 nm) 0.03 nA, −1.55 V, 4.8 K. f) High‐resolution STM images of a homochiral segment of ch‐GNR 9 corresponding to 10 NAP units. STM parameters: (7.7 nm×2.3 nm) 0.04 nA, −1.2 V, 4.8 K.

Further annealing the substrate at 570 K for 90 minutes induced the aryl‐aryl coupling and the cyclodehydrogenation between the phenyl and naphthalene rings, yielding **9**, as demonstrated by STM (Figure 2e and f). This reaction sequence is common for most of the previous on‐surface syntheses of GNRs and considered to proceed through 1) homolytic cleavage of carbon‐halogen bonds to generate diradical species stabilized by the metal surface; 2) radical polymerization of the diradical species that are mobile on the surface; and 3) oxidative cyclodehydrogenation catalysed by the metal surface.[^1a^, ^1e^, ^12a^] These ribbons are composed of relatively short segments of **9** connected through kinks. We attribute these kinks to defects caused by a lack of enantiomeric selectivity during the polymerization step. The high resolution STM image in Figure 2f shows a defect‐free, homochiral segment of the obtained GNR corresponding to 10 units of NAP (see superimposed scaled chemical structure), thus corroborating the formation of ch‐GNR **9** and elucidating its non‐planar structure induced by the presence of cove edges. Similar bright protrusions were reported for cove‐edge GNRs in ref. [7a] and attributed to steric repulsion between hydrogens atoms of benzene rings at the cove region.

The electronic properties of the obtained ch‐GNR **9** were characterized by means of STS. Figure 3a displays the single‐point differential conductance (*dI/dV*) spectra acquired at the edge of ch‐GNR **9** (blue and red curves, see coloured crosses in the inset STM image for the exact location of the measurements), together with the reference spectrum of the Au(111) showing its characteristic surface state. Both single‐point spectra reveal the appearance of two states with onsets at −0.5 V and 1.1 V, which can be attributed to the top of the valence band (VB) and the bottom of the conduction band (CB) of **9**, respectively. These values result in an electronic band gap (E_g_) of approximately 1.6 eV. To further corroborate the onset energies and the assignment of the corresponding states, we have recorded differential‐conductance maps at these energies. The spatial distribution of these states indeed corresponds to the onset of states located on the GNR (see Figure S2 for the complete set of *dI/dV* maps).

**Figure 3:**
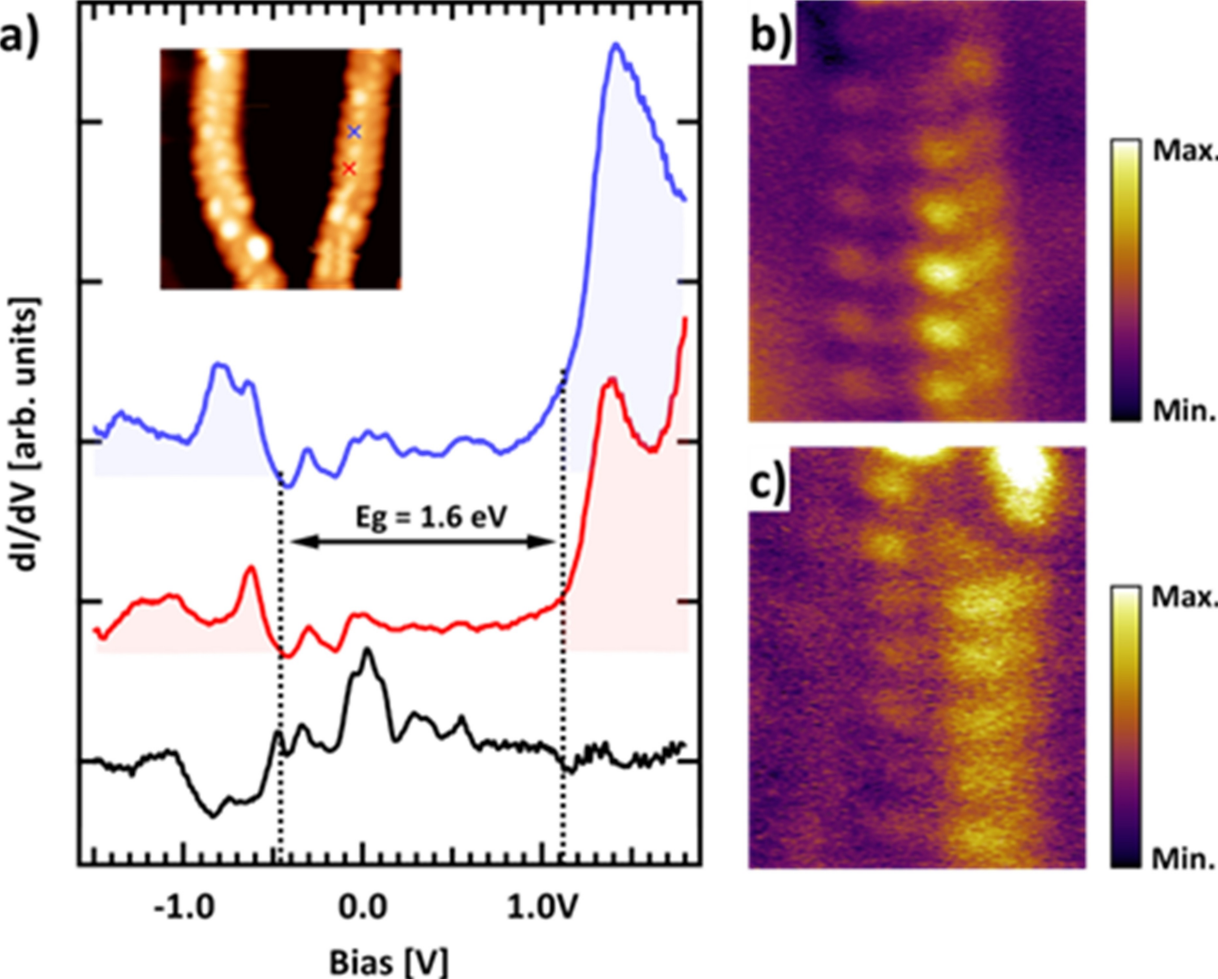
Electronic characterization of the ch‐GNR **9** by STS. a) Single‐point differential conductance (*dI/dV*) spectra measured at the GNR edge (blue and red) and on the Au(111) substrate. Dashed black lines indicate the onsets of the VB and CB of the GNRs. Inset: STM image showing the GNR where the spectra have been acquired (blue and red crosses). STM parameters: (7.7 nm×7.7 nm) 0.8 nA, −1.5 V. b, c) Differential‐conductance maps of occupied and unoccupied states taken at a sample bias of −0.5 V and 1.1 V, respectively. *dI/dV* map parameters: (3.2 nm×4.0 nm) 0.2 nA.

In conclusion, NAP **7** was synthesized as the precursor monomer, which allowed for the fabrication of unprecedented benzo‐fused chiral (2,1)‐GNR **9** having cove edges, on a Au (111) surface under UHV conditions. Characterization by STM evidenced the formation of **9** with a non‐planar structure originating from the cove edge, and an STS analysis revealed its relatively small bandgap of ∼1.6 eV. The obtained GNRs featured connections of segments with different chirality with defective structures at the junctions, due to the lack of enantiomeric selectivity along the ribbons during the polymerization and cyclodehydrogenation. This work expands the structural variety of GNRs, providing an insight into the non‐planarity induced by the cove edge geometry and formation of chiral GNRs. Further efforts to control the chirality of GNRs in the on‐surface synthesis are ongoing in our laboratories.

**Crystallographic data**: Deposition number **2021698** contains the supplementary crystallographic data for compound NAP **7**. This data are provided free of charge by the joint Cambridge Crystallographic Data Centre and Fachinformationszentrum Karlsruhe Access Structures service. Please see the Supporting Information for more details.

## Conflict of interest

The authors declare no conflict of interest.

## Supporting information

As a service to our authors and readers, this journal provides supporting information supplied by the authors. Such materials are peer reviewed and may be re‐organized for online delivery, but are not copy‐edited or typeset. Technical support issues arising from supporting information (other than missing files) should be addressed to the authors.

Supplementary

## References

[1a] A. Narita , Z. Chen , Q. Chen , K. Müllen , Chem. Sci. 2019, 10, 964–975; 30774890 10.1039/c8sc03780a PMC6349060

[1b] A. Narita , X.-Y. Wang , X. Feng , K. Mullen , Chem. Soc. Rev. 2015, 44, 6616–6643; 26186682 10.1039/c5cs00183h

[1c] M. Slota , A. Keerthi , W. K. Myers , E. Tretyakov , M. Baumgarten , A. Ardavan , H. Sadeghi , C. J. Lambert , A. Narita , K. Müllen , L. Bogani , Nature 2018, 557, 691–695; 29849157 10.1038/s41586-018-0154-7

[1d] A. Bianco , Y. Chen , Y. Chen , D. Ghoshal , R. H. Hurt , Y. A. Kim , N. Koratkar , V. Meunier , M. Terrones , Carbon 2018, 132, 785–801;

[1e] X. Zhou , G. Yu , Adv. Mater. 2020, 32, 1905957; 10.1002/adma.201905957 31830353

[1f] Y. Yano , N. Mitoma , H. Ito , K. Itami , J. Org. Chem. 2020, 85, 4–33; 31789025 10.1021/acs.joc.9b02814

[1g] F. Bonaccorso , Z. Sun , T. Hasan , A. C. Ferrari , Nat. Photonics 2010, 4, 611–622;

[1h] Z. Geng , B. Hähnlein , R. Granzner , M. Auge , A. A. Lebedev , V. Y. Davydov , M. Kittler , J. Pezoldt , F. Schwierz , Ann. Phys. 2017, 529, 1700033;

[1i] K. Sun , X. Li , L. Chen , H. Zhang , L. Chi , J. Phys. Chem. C 2020, 124, 11422–11427;

[1j] M. Mehdi Pour , A. Lashkov , A. Radocea , X. Liu , T. Sun , A. Lipatov , R. A. Korlacki , M. Shekhirev , N. R. Aluru , J. W. Lyding , V. Sysoev , A. Sinitskii , Nat. Commun. 2017, 8, 820; 29018185 10.1038/s41467-017-00692-4 PMC5635063

[1k] S. von Kugelgen , I. Piskun , J. H. Griffin , C. T. Eckdahl , N. N. Jarenwattananon , F. R. Fischer , J. Am. Chem. Soc. 2019, 141, 11050–11058. 31264864 10.1021/jacs.9b01805

[2a] O. Gröning , S. Wang , X. Yao , C. A. Pignedoli , G. Borin Barin , C. Daniels , A. Cupo , V. Meunier , X. Feng , A. Narita , K. Müllen , P. Ruffieux , R. Fasel , Nature 2018, 560, 209–213; 30089919 10.1038/s41586-018-0375-9

[2b] I. Ivanov , Y. Hu , S. Osella , U. Beser , H. I. Wang , D. Beljonne , A. Narita , K. Müllen , D. Turchinovich , M. Bonn , J. Am. Chem. Soc. 2017, 139, 7982–7988; 28525278 10.1021/jacs.7b03467

[2c] A. Keerthi , B. Radha , D. Rizzo , H. Lu , V. Diez Cabanes , I. C.-Y. Hou , D. Beljonne , J. Cornil , C. Casiraghi , M. Baumgarten , K. Müllen , A. Narita , J. Am. Chem. Soc. 2017, 139, 16454–16457; 29098859 10.1021/jacs.7b09031 PMC5860787

[2d] O. V. Yazyev , Acc. Chem. Res. 2013, 46, 2319–2328; 23282074 10.1021/ar3001487

[2e] A. Celis , M. N. Nair , A. Taleb-Ibrahimi , E. H. Conrad , C. Berger , W. A. de Heer , A. Tejeda , J. Phys. D 2016, 49, 143001;

[2f] T. H. Vo , M. Shekhirev , D. A. Kunkel , M. D. Morton , E. Berglund , L. Kong , P. M. Wilson , P. A. Dowben , A. Enders , A. Sinitskii , Nat. Commun. 2014, 5, 3189; 24510014 10.1038/ncomms4189

[2g] J. D. Teeter , P. Zahl , M. Mehdi Pour , P. S. Costa , A. Enders , A. Sinitskii , ChemPhysChem 2019, 20, 2281–2285; 31185134 10.1002/cphc.201900445

[2h] M. Shekhirev , P. Zahl , A. Sinitskii , ACS Nano 2018, 12, 8662–8669; 30085655 10.1021/acsnano.8b04489

[2i] D. J. Rizzo , G. Veber , T. Cao , C. Bronner , T. Chen , F. Zhao , H. Rodriguez , S. G. Louie , M. F. Crommie , F. R. Fischer , Nature 2018, 560, 204–208. 30089918 10.1038/s41586-018-0376-8

[3] T. Cao , F. Zhao , S. G. Louie , Phys. Rev. Lett. 2017, 119, 076401. 28949674 10.1103/PhysRevLett.119.076401

[4a] C. Tao , L. Jiao , O. V. Yazyev , Y.-C. Chen , J. Feng , X. Zhang , R. B. Capaz , J. M. Tour , A. Zettl , S. G. Louie , H. Dai , M. F. Crommie , Nat. Phys. 2011, 7, 616–620;

[4b] M. Ezawa , Phys. Rev. B 2006, 73, 045432.

[5] O. V. Yazyev , R. B. Capaz , S. G. Louie , Phys. Rev. B 2011, 84, 115406.

[6] Y. Yano , N. Mitoma , K. Matsushima , F. Wang , K. Matsui , A. Takakura , Y. Miyauchi , H. Ito , K. Itami , Nature 2019, 571, 387–392. 31243361 10.1038/s41586-019-1331-z

[7a] J. Liu , B.-W. Li , Y.-Z. Tan , A. Giannakopoulos , C. Sanchez-Sanchez , D. Beljonne , P. Ruffieux , R. Fasel , X. Feng , K. Müllen , J. Am. Chem. Soc. 2015, 137, 6097–6103; 25909566 10.1021/jacs.5b03017 PMC4456008

[7b] Y. Yano , F. Wang , N. Mitoma , Y. Miyauchi , H. Ito , K. Itami , J. Am. Chem. Soc. 2020, 142, 1686–1691; 31918548 10.1021/jacs.9b11328

[7c] N. Mitoma , Y. Yano , H. Ito , Y. Miyauchi , K. Itami , ACS Appl. Nano Mater. 2019, 2, 4825–4831.

[8a] W. Xu , T.-W. Lee , Mater. Horiz. 2016, 3, 186–207;

[8b] H. Shen , Y. Shi , X. Wang , Synth. Met. 2015, 210, 109–122.

[9] X.-Y. Wang , A. Narita , K. Müllen , Nat. Chem. Rev. 2017, 2, 0100.

[10a] A. Narita , X. Feng , Y. Hernandez , S. A. Jensen , M. Bonn , H. Yang , I. A. Verzhbitskiy , C. Casiraghi , M. R. Hansen , A. H. R. Koch , G. Fytas , O. Ivasenko , B. Li , K. S. Mali , T. Balandina , S. Mahesh , S. De Feyter , K. Müllen , Nat. Chem. 2014, 6, 126–132; 24451588 10.1038/nchem.1819

[10b] K.-Y. Yoon , G. Dong , Mater. Chem. Front. 2020, 4, 29–45;

[10c] A. Jolly , D. Miao , M. Daigle , J.-F. Morin , Angew. Chem. Int. Ed. 2020, 59, 4624–4633. 10.1002/anie.201906379 31265750

[11a] P. Ruffieux , S. Wang , B. Yang , C. Sánchez-Sánchez , J. Liu , T. Dienel , L. Talirz , P. Shinde , C. A. Pignedoli , D. Passerone , T. Dumslaff , X. Feng , K. Müllen , R. Fasel , Nature 2016, 531, 489–492; 27008967 10.1038/nature17151

[11b] X. Li , H. Zhang , L. Chi , Adv. Mater. 2019, 31, 1804087; 10.1002/adma.201804087 30592340

[11c] H. Zhang , H. Lin , K. Sun , L. Chen , Y. Zagranyarski , N. Aghdassi , S. Duhm , Q. Li , D. Zhong , Y. Li , K. Müllen , H. Fuchs , L. Chi , J. Am. Chem. Soc. 2015, 137, 4022–4025; 25775004 10.1021/ja511995r

[11d] S. Clair , D. G. de Oteyza , Chem. Rev. 2019, 119, 4717–4776. 30875199 10.1021/acs.chemrev.8b00601 PMC6477809

[12a] L. Talirz , P. Ruffieux , R. Fasel , Adv. Mater. 2016, 28, 6222–6231; 26867990 10.1002/adma.201505738

[12b] M. Kolmer , A. K. Steiner , I. Izydorczyk , W. Ko , M. Engelund , M. Szymonski , A.-P. Li , K. Amsharov , Science 2020, 369, eabb8880. 10.1126/science.abb8880 32586951

[13] O. Deniz , C. Sánchez-Sánchez , T. Dumslaff , X. Feng , A. Narita , K. Müllen , N. Kharche , V. Meunier , R. Fasel , P. Ruffieux , Nano Lett. 2017, 17, 2197–2203. 28301723 10.1021/acs.nanolett.6b04727

[14a] N. Merino-Díez , M. S. G. Mohammed , J. Castro-Esteban , L. Colazzo , A. Berdonces-Layunta , J. Lawrence , J. I. Pascual , D. G. de Oteyza , D. Peña , Chem. Sci. 2020; 10.1039/d0sc01653e PMC8159356 34094071

[14b] C. Sánchez-Sánchez , T. Dienel , O. Deniz , P. Ruffieux , R. Berger , X. Feng , K. Müllen , R. Fasel , ACS Nano 2016, 10, 8006–8011; 27428831 10.1021/acsnano.6b04025

[14c] P. Han , K. Akagi , F. Federici Canova , H. Mutoh , S. Shiraki , K. Iwaya , P. S. Weiss , N. Asao , T. Hitosugi , ACS Nano 2014, 8, 9181–9187; 25162921 10.1021/nn5028642

[14d] P. Han , K. Akagi , F. Federici Canova , R. Shimizu , H. Oguchi , S. Shiraki , P. S. Weiss , N. Asao , T. Hitosugi , ACS Nano 2015, 9, 12035–12044; 26588477 10.1021/acsnano.5b04879

[14e] D. G. de Oteyza , A. Garcia-Lekue , M. Vilas-Varela , N. Merino-Diez , E. Carbonell-Sanroma , M. Corso , G. Vasseur , C. Rogero , E. Guitian , J. I. Pascual , J. E. Ortega , Y. Wakayama , D. Pena , ACS Nano 2016, 10, 9000–9008; 27548516 10.1021/acsnano.6b05269 PMC5043421

[14f] F. Schulz , P. H. Jacobse , F. F. Canova , J. van der Lit , D. Z. Gao , A. van den Hoogenband , P. Han , R. J. M. Klein Gebbink , M.-E. Moret , P. M. Joensuu , I. Swart , P. Liljeroth , J. Phys. Chem. C 2017, 121, 2896–2904.

[15] X.-Y. Wang , J. I. Urgel , G. B. Barin , K. Eimre , M. Di Giovannantonio , A. Milani , M. Tommasini , C. A. Pignedoli , P. Ruffieux , X. Feng , R. Fasel , K. Müllen , A. Narita , J. Am. Chem. Soc. 2018, 140, 9104–9107. 29990420 10.1021/jacs.8b06210

[16] J.-Y. Wang , Y. Zhou , J. Yan , L. Ding , Y. Ma , Y. Cao , J. Wang , J. Pei , Chem. Mater. 2009, 21, 2595–2597.

[17] R. Gutzler , H. Walch , G. Eder , S. Kloft , W. M. Heckl , M. Lackinger , Chem. Commun. 2009, 4456–4458. 10.1039/b906836h 19597624

[18] L. Talirz , H. Söde , T. Dumslaff , S. Wang , J. R. Sanchez-Valencia , J. Liu , P. Shinde , C. A. Pignedoli , L. Liang , V. Meunier , N. C. Plumb , M. Shi , X. Feng , A. Narita , K. Müllen , R. Fasel , P. Ruffieux , ACS Nano 2017, 11, 1380–1388. 28129507 10.1021/acsnano.6b06405

