# Supplementary material for: On‐surface Synthesis of a Chiral Graphene Nanoribbon with Mixed Edge Structure
Source: Chem Asian J. 2020 Oct 12;15(22):3807–11. doi: 10.1002/asia.202001008 (PMC7756733; doi:10.1002/asia.202001008)
Supplement: Supplementary file 1 — Supplementary [file ASIA-15-3807-s001.pdf]

CHEMISTRY
AN ASIAN JOURNAL

Supporting Information

On-surface Synthesis of a Chiral Graphene Nanoribbon with Mixed Edge Structure

Ashok Keerthi[+], Carlos Sánchez-Sánchez[+], Okan Deniz, Pascal Ruffieux, Dieter Schollmeyer, Xinliang Feng, Akimitsu Narita, Roman Fasel,* and Klaus Müllen*This manuscript is part of a special collection for the 20th Anniversary of the Tateshina Conference.© 2020 The Authors. Published by Wiley-VCH GmbH. This is an open access article under the terms of the Creative Commons Attribution License, which permits use, distribution and reproduction in any medium, provided the original work is properly cited.

Contents:

1. Materials and methods

2. Experimental Details

3. Single crystal XRD data of compound 7

4. STM images and *dI/dV* maps

5. NMR spectra

6. Reference

1. Materials and methods:

1.1. Solution synthesis

All chemicals and reagents were purchased from commercial suppliers and used without further purification. Solvents used for spectroscopic measurements were spectral grade quality. All other chemicals, reagents and 2,6-dimethoxynaphthalene (**1**) were purchased from commercials suppliers and used as received. All reactions were monitored by thin-layer chromatography (TLC) carried out on silica gel plates. Preparative separations were performed by column chromatography on silica gel grade 60 (0.040 - 0.063 mm) from Merck.

^{1}H and ^{13}C NMR spectra were recorded in deuterated solvents such as CD_2Cl_2, using a spectrometers Bruker Avance 300 MHz and Bruker DPX 250. The chemical shifts were reported in ppm and referenced to the residual solvent peak. s = singlet, d = doublet, t = triplet, m = multiplet, b = broad. Field desorption (FD) mass spectra (MS) were performed with a VG-Instrument ZAB 2-SE-FDP.

1.2. UHV synthesis

On-surface experiments have been carried out in two ultrahigh vacuum (UHV) chambers at the Nanotech@surface group (Empa). One is equipped with a low-temperature scanning tunneling microscopy (STM) while the other includes low-temperature scanning tunneling microscopy and non-contact atomic force microscopy (STM/nc-AFM) (Scienta Omicron). Au(111) single crystals were prepared by repeated cycles of sputtering with Ar^{+} (1.0 kV) and annealing (750 K) until judged clean by STM. Precursor monomer **7** was sublimed under UHV conditions using a home-made 6-fold evaporator at a temperature of 390 K (1 ML in 2 minutes). STM images were acquired in the constant-current mode at the temperature indicated in each figure caption. d*I*/d*V* spectra and maps were measured using the lock-in technique (860 Hz, 20 mV) at constant current mode.

2. Experimental Details:

2.1. Synthesis of 1,5-dibromo-2,6-dimethoxynaphthalene (2)[1]

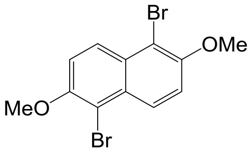

Compound **2** was synthesized by following a reported procedure.[1] To a Schlenk tube was added 2,6-dimethoxynaphthalene (**1**) (1.88 g, 10.0 mmol) in a mixture of $CHCl_3$ and acetic acid (1:1, 150 mL) was added N-Bromosuccinimide (NBS) (3.80 g, 21.3 mmol) in small portions at 0 °C. After stirring for 10 hours at room temperature, the mixture was quenched

with a saturated $NaHCO_3$ solution and extracted with $CHCl_3$. The combined organic layers were washed with brine and dried over Na_2SO_4. The solvent was removed in vacuo and the crude product was purified by column chromatography on silica gel using hexane as an eluent to give the title compound as a white solid (3.39 g, 98% yield). ^1H NMR (CD_2Cl_2, 300 MHz): δ (ppm) = 8.24 (2H, d, J = 9.3 Hz), 7.38 (2H, d, J = 9.3 Hz), 4.01 (3H, s). ^{13}C NMR (CD_2Cl_2, 75 MHz): δ (ppm) = 153.0, 129.0, 128.1, 115.0, 108.9, 57.3. MS (FD): calc.: 346.0 found: 345.8. ^1H and ^{13}C NMR results agreed with the previous report.[1]

2.2. Synthesis of 1,5-diphenyl-2,6-dimethoxynaphthalene (**3**)

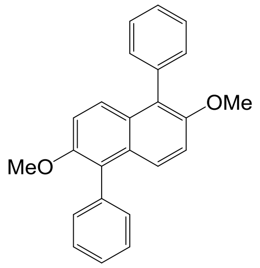

To a 250-mL two-neck round-bottom flask were added 1,5-dibromo-2,6-dimethoxylnaphthalene (1.73 g, 5.0 mmol), phenylboronic acid (1.23 g, 10.1 mmol), and K_2CO_3 (6.91 g, 50.0 mmol), toluene (120 mL), water (25 mL), and ethanol (12 mL). The reaction mixture was then degassed by argon bubbling for 30 minutes at room temperature, and then $Pd(PPh_3)_4$ (0.30 g, 0.26 mmol) was added. After stirring at 90 °C for 12 hours under argon atmosphere, the reaction mixture was allowed to cool to room temperature and was extracted with diethyl ether. The combined organic layers were washed with brine, dried over anhydrous sodium sulfate, and dried in vacuo. The crude product was purified by silica gel column chromatography using hexane as eluent to give the title compound as white solid (1.53 g, 90% yield). ^1H NMR (250 MHz, CD_2Cl_2): δ (ppm) = 7.56 - 7.48 (m, 8H), 7.42 - 7.39 (m, 4H), 7.29 (d, J = 8 Hz, 2H), 3.82 (s, 6H). ^{13}C NMR (62.5 MHz, CD_2Cl_2): δ (ppm) = 152.70, 137.38, 131.58, 129.70, 128.71, 127.60, 126.89, 125.69, 114.99, 57.21. MS (FD): calc.: 340.4 found: 340.3.

2.3. Synthesis of 1,5-diphenyl-2,6-dihydroxynaphthalene (**4**)

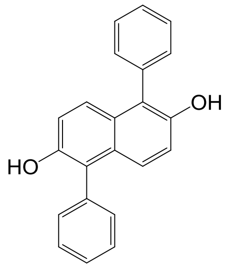

To a solution of **3** (1.7 g, 5.0 mmol) in 120 mL of dry dichloromethane (DCM) was added BBr_3 (1.4 mL, 14.7 mmol) dropwise at 0 °C. The solution was then slowly warmed to room temperature and stirred for 12 h. After quenching the reaction by adding water, the organic layer was washed with brine and dried over anhydrous Na_2SO_4. The solvent was removed in vacuo and the crude product was purified by silica gel column chromatography using hexane/DCM (1:1 ratio) as eluent to give compound **4** as a white solid (1.48 g, 95% yield).

^{1}H NMR (300 MHz, CD_2Cl_2) δ (ppm) = 7.63 - 7.59 (m, 4H), 7.54 (d, J = 7.2 Hz, 2H), 7.44 (d, J = 8.1 Hz, 4H), 7.35 (d, J = 9.0 Hz, 2H), 7.10 (d, J = 9.0 Hz, 2H), 5.03 (s, 2H). ^{13}C NMR (75 MHz, CD_2Cl_2) δ (ppm) = 149.00, 135.00, 131.73, 130.12, 129.17, 128.99, 126.63, 122.08, 118.24. MS (FD): calc.: 312.4 found: 312.1.

2.4. 1,5-diphenylnaphthalene-2,6-diyl bis(trifluoromethanesulfonate) (**5**)

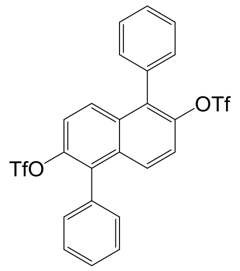

To a 100-mL two-neck round-bottom flask under nitrogen atmosphere, compound **4** (1.56 g, 5.0 mmol) and trimethylamine (3.0 mL, 21.6 mmol) were added and dissolved in DCM (50 mL). The reaction mixture was cooled to 0 °C and trifluoromethanesulfonic anhydride (1.5 mL, 8.9 mmol) was added. The reaction mixture was stirred at 50 °C for 10 hours under nitrogen atmosphere. The reaction was quenched with water and 1M HCl solution and extracted with DCM. The combined organic layers were washed with saturated aqueous $NaHCO_3$ solution and brine and then dried over Na_2SO_4. The solvent was removed in vacuo and the crude product was purified by column chromatography on silica gel using hexane/ethyl acetate (10:1) as an eluent to give the title compound as a white solid (2.51 g, 87% yield). ^{1}H NMR (250 MHz, CD_2Cl_2) δ (ppm) = 7.78 (d, J = 9.0 Hz, 2H), 7.60 - 7.57 (m, 6H), 7.51 - 7.42 (m, 6H). ^{13}C NMR (62.5 MHz, CD_2Cl_2) δ (ppm) = 145.50, 133.57, 133.40, 132.99, 131.31, 129.58, 129.52, 129.19, 121.51, 116.33. MS (FD): calc.: 576.5 found: 576.1.

2.5. Synthesis of 2,2'-(1,5-diphenylnaphthalene-2,6-diyl)bis(4,4,5,5-tetramethyl-1,3,2-dioxaborolane) (6)

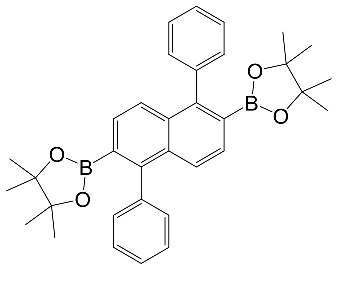

Compound **5** (2.02 g, 3.5 mmol) and pinacolborane (1.5 g, 10.3 mmol) were added to a 100-mL Schlenk tube and dissolved in 20 mL of dry dioxane. To this solution, KOAc (1.2 g, 12.2 mmol) was added, followed by degassing through argon bubbling for 30 mins. To the reaction mixture, Pd(dppf)Cl_2 (250 mg, 0.35 mmol) was added and the reaction mixture was refluxed overnight under nitrogen atmosphere. After cooling to room temperature, the reaction was quenched with water, followed by extraction with diethyl ether. The combined organic layers were washed with brine and dried over Na_2SO_4. After evaporation, the crude product was purified by column chromatography on silica gel using hexane/ethyl acetate (10:2) solvent mixture as an eluent to give compound **6** as a white solid (1.14 g, 61% yield). ^{1}H NMR (300 MHz, CD_2Cl_2) δ (ppm) = 7.61 (d, 4H), 7.46 - 7.42 (m, 6H), 7.37 - 7.33 (m,

4H), 1.10 (s, 24H). ^{13}C NMR (62.5 MHz, CD_2Cl_2) δ (ppm) = 146.80, 141.86, 133.33, 131.14, 129.94, 128.13, 127.52, 125.84, 84.12, 24.87. MS (FD): calc.: 532.3 found: 532.4.

2.6. Synthesis of 2,6-dibromo-1,5-diphenylnaphthalene, (NAP 7)

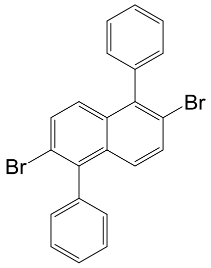

To a 100-mL two-neck round-bottom flask equipped with a condenser, compound **6** (0.53 g, 1.0 mmol) and $CuBr_2$ (0.70 g, 3.1 mmol) were added and dissolved in dioxane/MeOH/H_2O (30 mL, 1:1:1). The mixture was refluxed for 24 hours and cooled down to room temperature. The reaction mixture was extracted with DCM and the organic phase was washed with water and brine. After drying over Na_2SO_4, solvents were removed in vacuo. The crude product was purified by column chromatography on silica gel using hexane/DCM (3:1) solvent mixture as an eluent to give compound **7, NAP** as a white solid (0.32 g, 73% yield). Recrystallization from DCM/MeOH gave needle-shaped colourless single crystals.^{1}H NMR (250 MHz, CD_2Cl_2) δ (ppm) = 7.63 - 7.53 (m, 8H), 7.33 - 7.30 (m, 6H). ^{13}C NMR (62.5 MHz, CD_2Cl_2) δ (ppm) = 140.47, 139.84, 133.47, 131.27, 130.61, 129.05, 128.57, 128.31, 122.38. MS (FD): calc.: 438.2 found: 438.0.

3. Single crystal analysis of compound 7 – CCDC Deposition Number 2021698

	Crystal data		
Formula	$C_{22}H_{14}Br_2$		
Molecular weight	438.15 $gmol^{-1}$		
Absorption	$\mu = 4.733\ mm^{-1}$ correction with 6 crystal faces		
Transmission	$t_{min} = 0.2828$, $t_{max} = 0.6332$		
Crystal size	0.12 x 0.12 x 0.29 mm^3 colourless needle		
Space group	P -1 (triclinic)		
Lattice parameters	a = 7.9199(7)Å	α = 104.661(7)°	
(calculate from	b = 9.4727(9)Å	β = 90.371(7)°	
12738 reflections with	c = 12.2490(10)Å	γ = 105.017(7)°	
2.49° < θ < 28.31°)	V = 856.12(14) $Å^3$	z = 2	F(000) = 432
Temperature	-80 °C		
Density	$d_{xray} = 1.700\ gcm^{-3}$		
	Data collection		
Diffractometer	STOE IPDS 2T		
Radiation	Mo-K_α Graphitmonochromator		
Scan – type	ω scans		
Scan – width	1°		
Scan range	$2° \leq \theta < 28°$		
	$-10 \leq h \leq 10$ $-12 \leq k \leq 12$ $-16 \leq l \leq 16$		
Number of reflections:			
measured	7964		
Unique	4182 (R_{int} = 0.0403)		

Observed	3133 ($\|F\|/\sigma(F) > 4.0$)

<u>Data correction, structure solution and refinement</u>

Corrections	Lorentz and polarisation correction.
Structure solution	Program: SIR-2004 (Direct methods)
Refinement	Program: SHELXL-2014 (full matrix). 217 refined parameters, weighting scheme: $w=1/[\sigma^2(F_o^2) + (0.0623*P)^2+1.34*P]$ with $(Max(F_o^2,0)+2*F_c^2)/3$. H-atoms at calculated positions and refined with isotropic displacement parameters, non H- atoms refined anisotropically.
R-values	wR2 = 0.1272 (R1 = 0.0456 for observed reflections, 0.0654 for all reflections)
Goodness of fit	S = 1.03
Maximum deviation of parameters	0.001 * e.s.d
Maximum peak height in diff. Fourier synthesis	0.7, -1.21 eÅ^{-3}
remark	structure contains two independent molecules with C_i symmetry

4. STM image and *dI/dV* maps of ch-GNR:

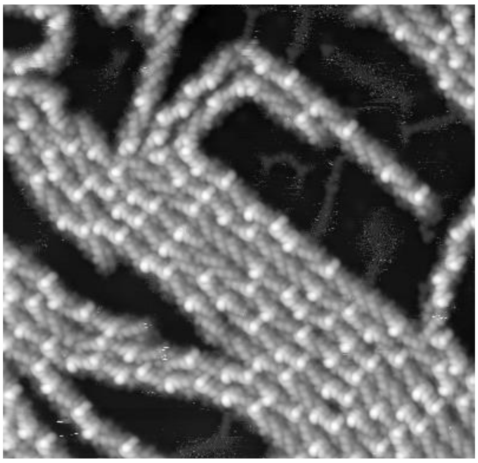

Figure S1. STM image of the metal-coordinated NAP molecular chains obtained after annealing the system at 470 K. The bright long chains correspond to the molecular chains while the faint irregular structures in between are ascribed to detached Br atoms on the Au(111) surface, thus indicating the dehalogenation of the molecules at this temperature. STM parameters: (30 nm × 30 nm) I = 40 pA, V = -1.3 V.

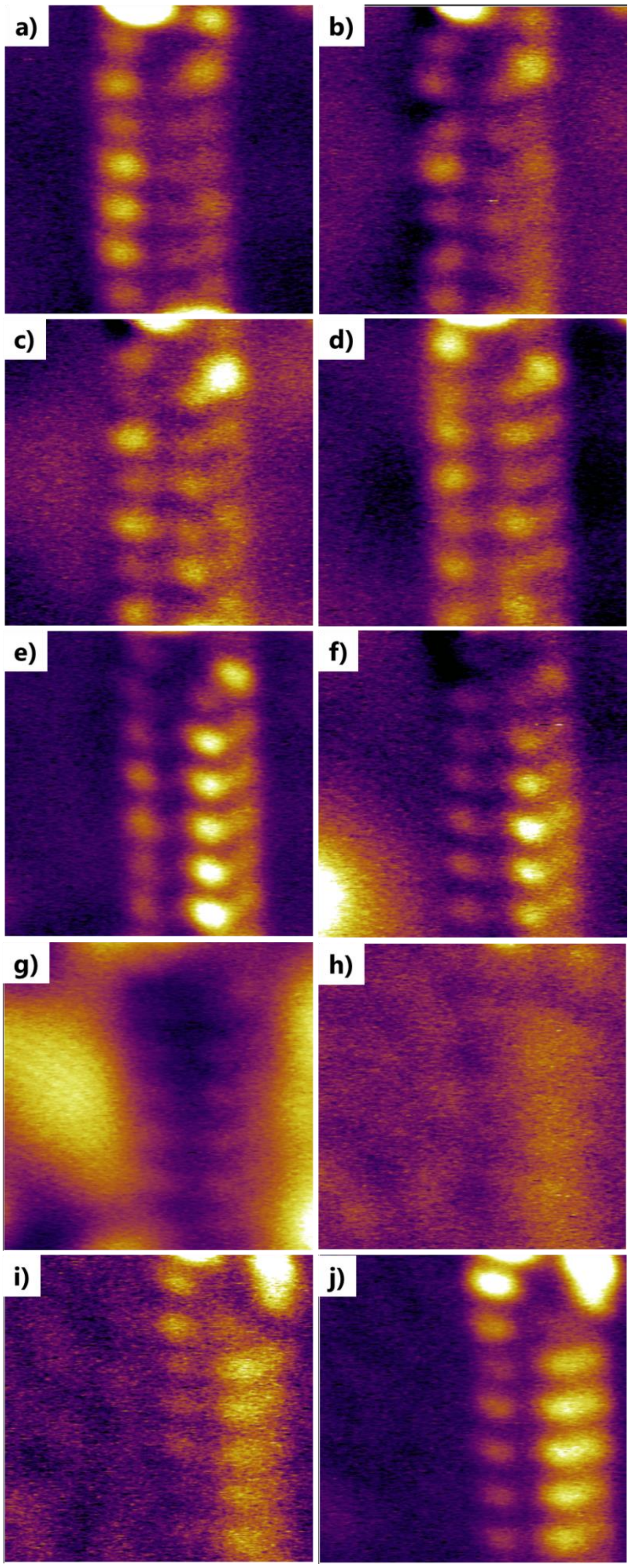

Figure S2. Set of dI/dV maps at **a)** –1.5 V, **b)** –1.3 V, **c)** –1.1 V, **d)** –0.9 V, **e)** –0.7 V, **f)** –0.5 V, **g)** –0.3 V, **h)** 0.9 V, **i)** 1.1 V, **j)** 1.3 V. Maps in between –0.3 V and 0.9 V have been omitted as there is no signal from the GNRs. STM parameters: (4 nm × 4 nm) I = 200 pA.

5. NMR spectra of synthesized compounds:

5.1. ^{1}H and ^{13}C NMR spectra of compound **1** (250 and 62.5 MHz, respectively, recorded in CD_2Cl_2)

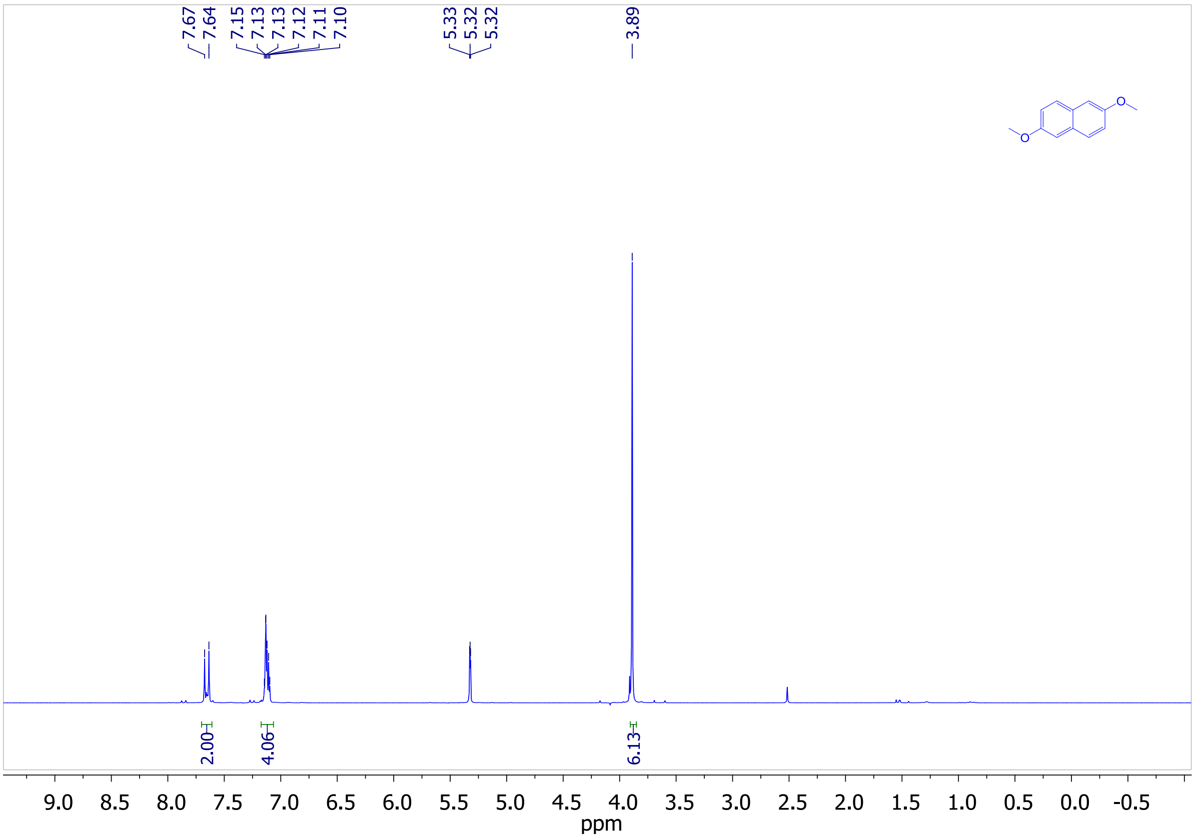

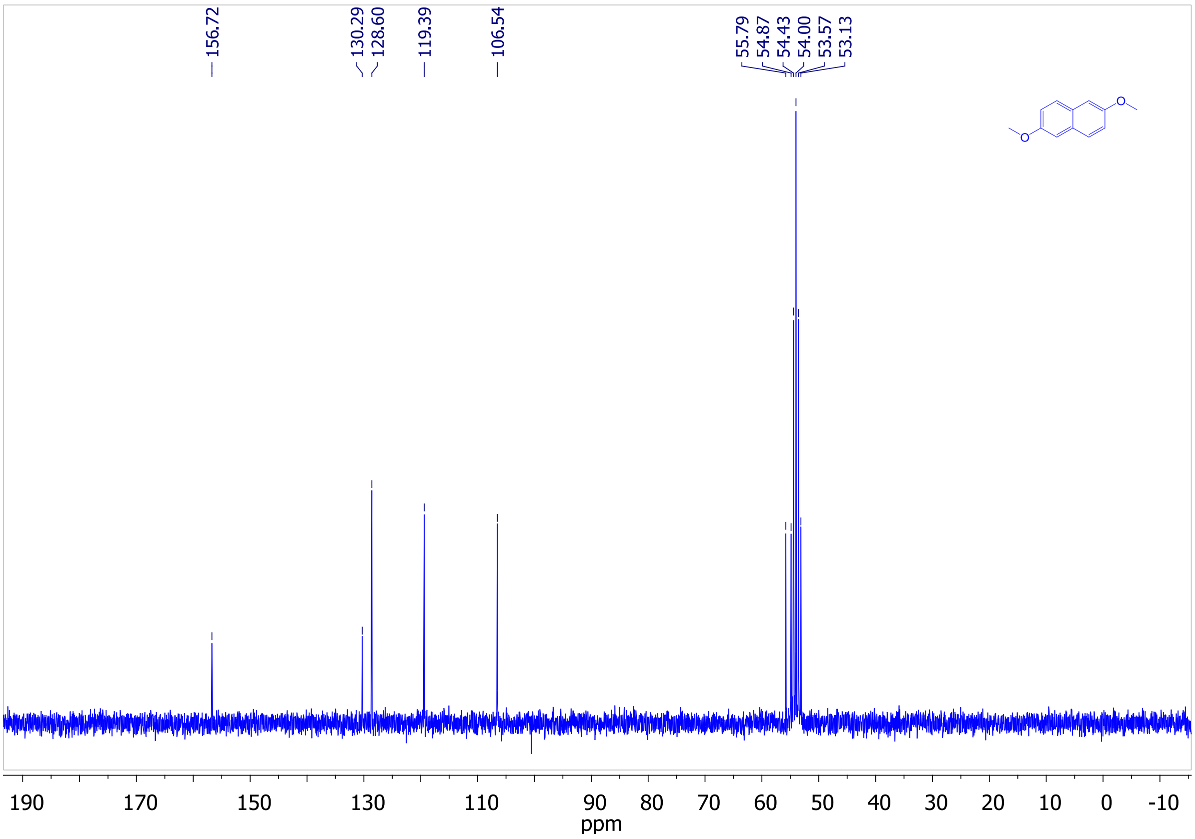

5.2. ^{1}H and ^{13}C NMR spectra of compound **2** (300 and 75 MHz, respectively, recorded in CD_2Cl_2)

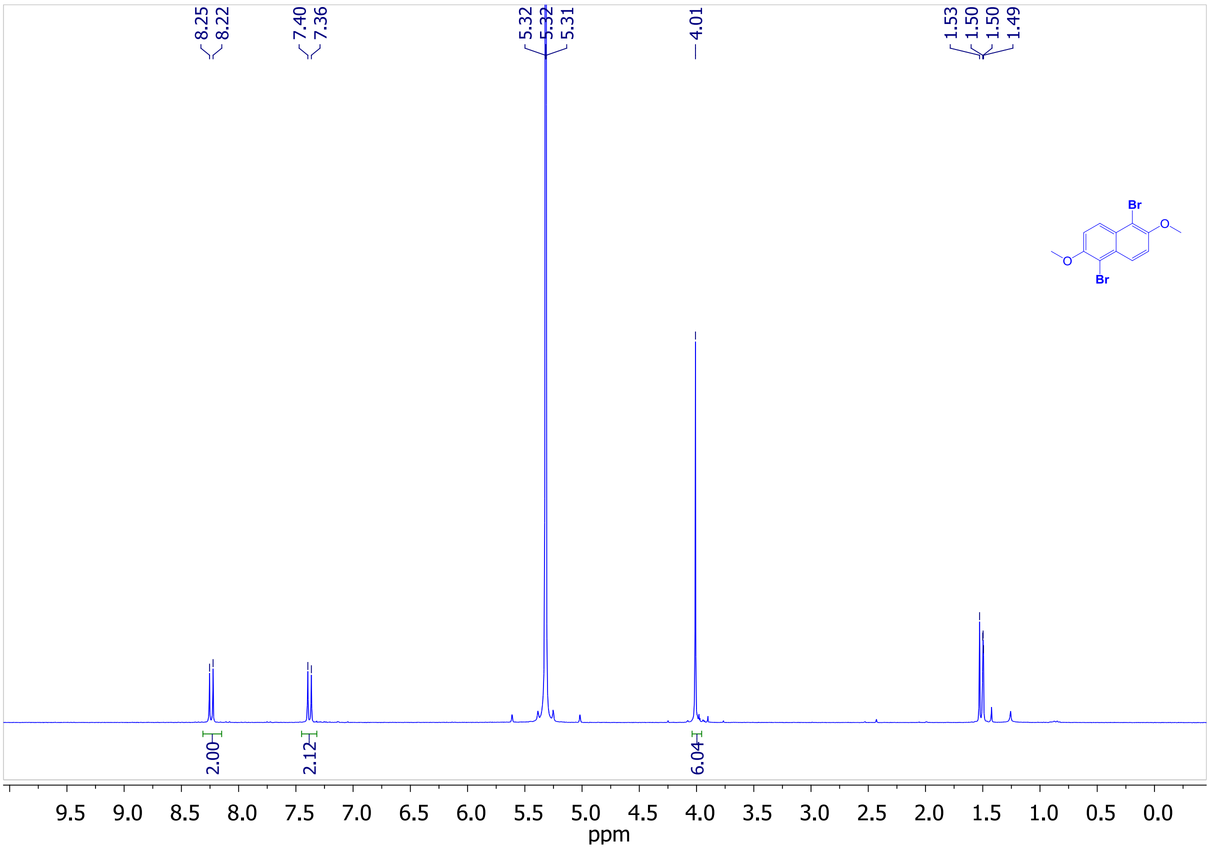

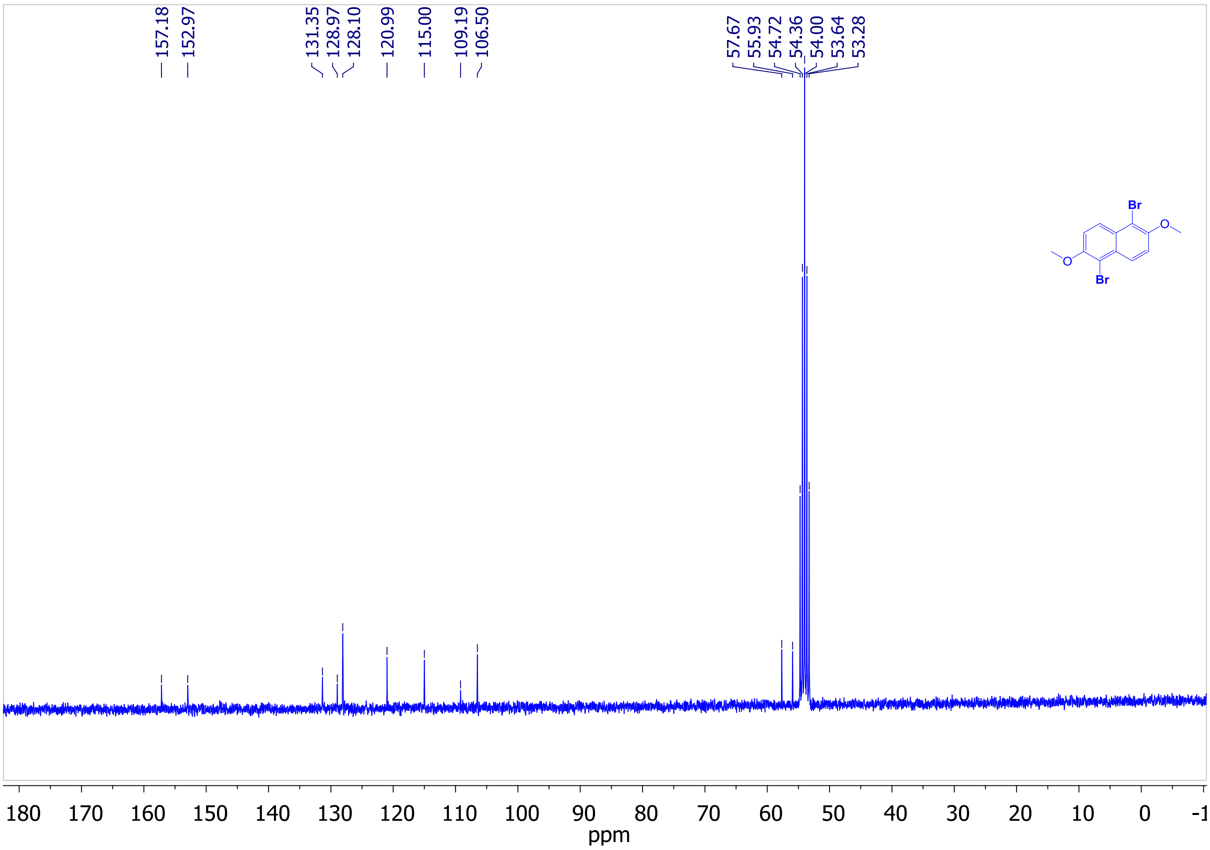

5.3. ^{1}H and ^{13}C NMR spectra of compound **3** (250 and 62.5 MHz, respectively, recorded in CD_2Cl_2)

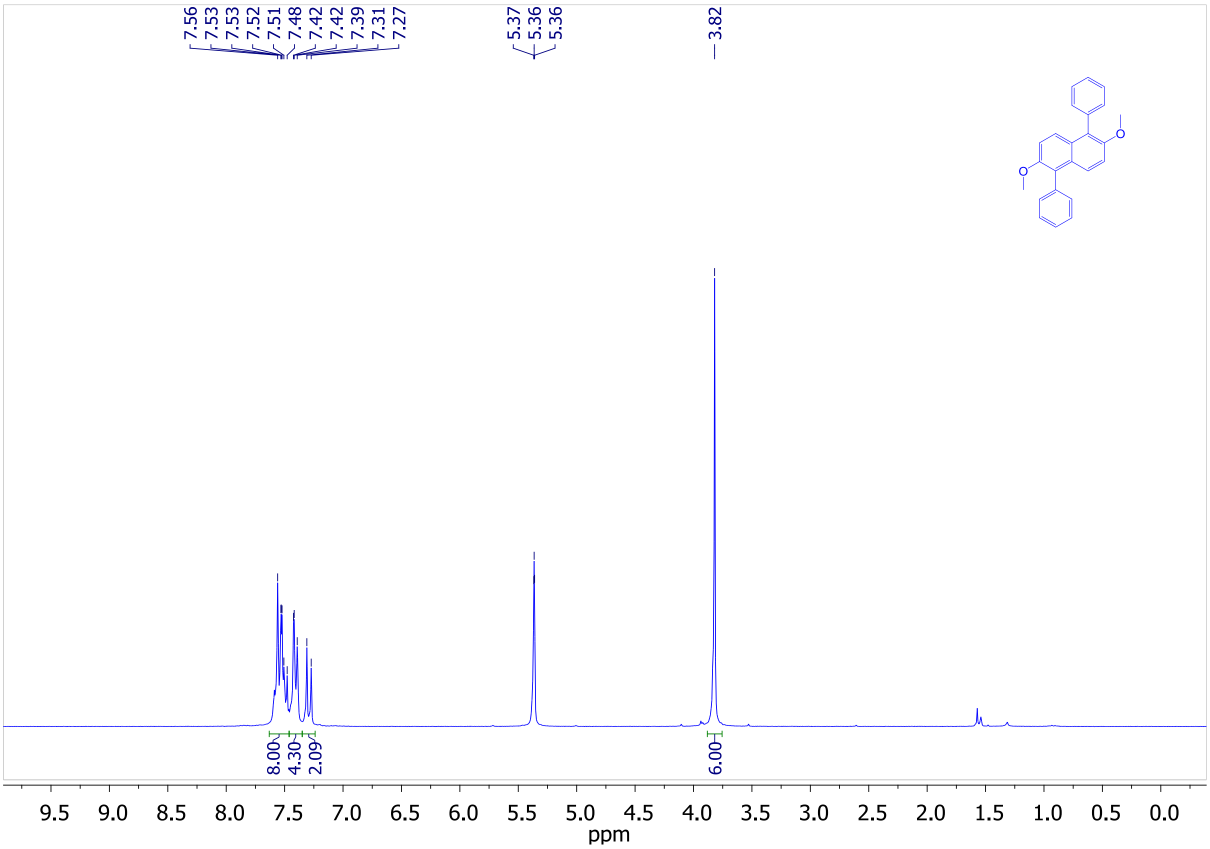

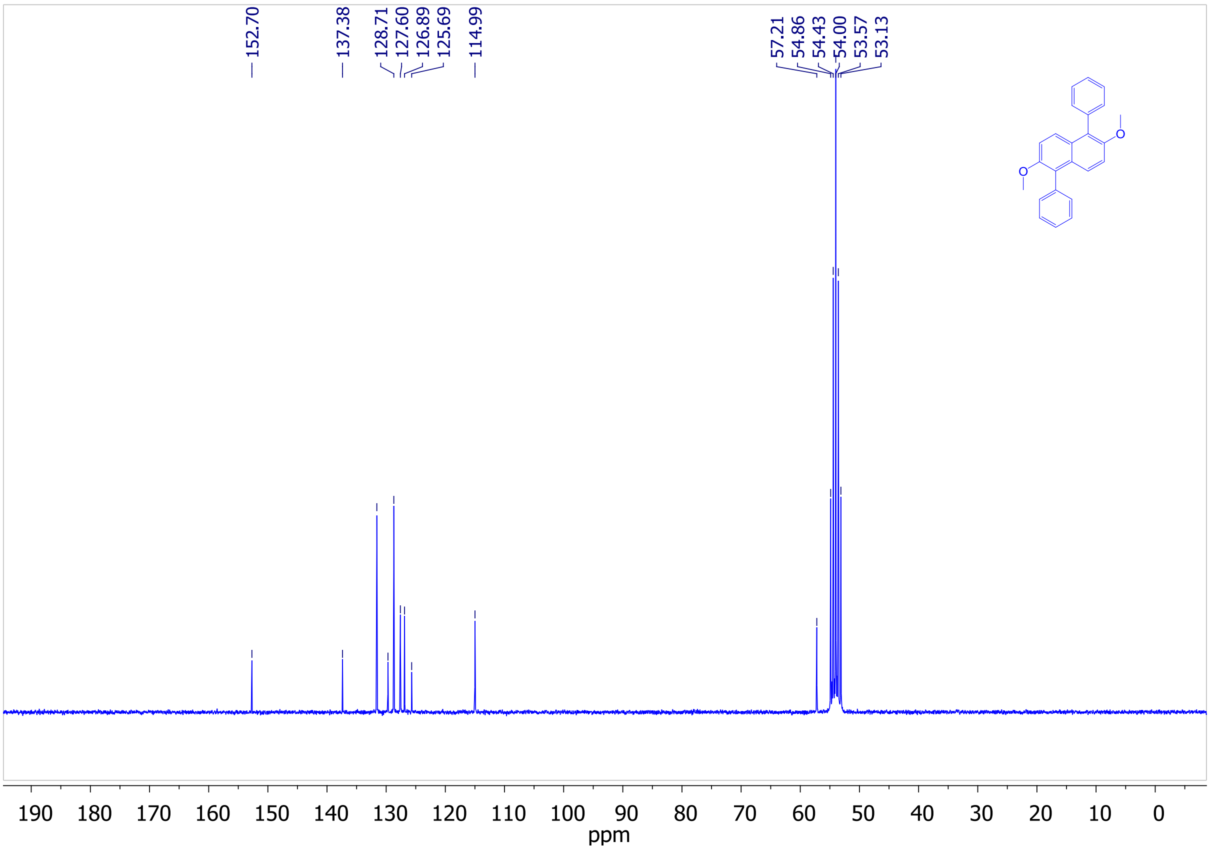

5.4. ^{1}H and ^{13}C NMR spectra of compound **4** (300 and 75 MHz, respectively, recorded in CD_2Cl_2)

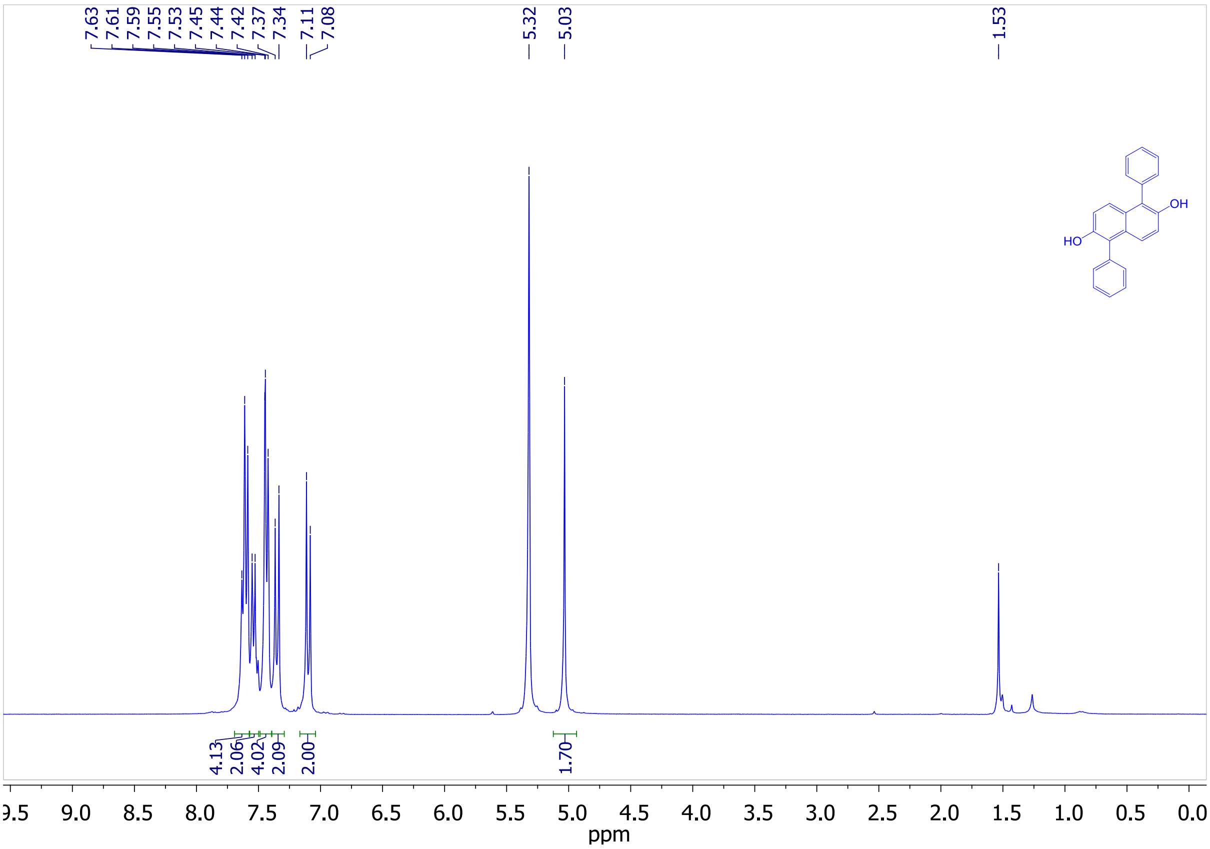

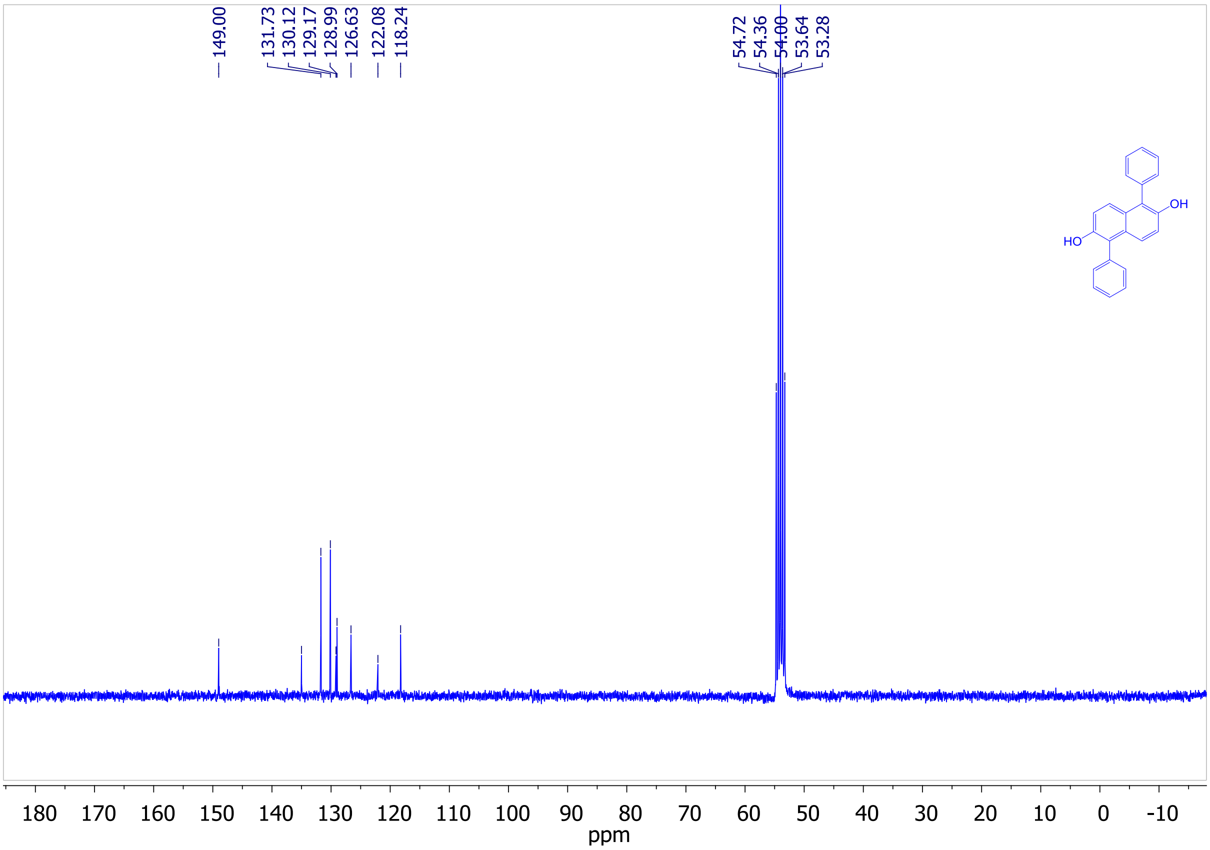

5.5. ^{1}H and ^{13}C NMR spectra of compound **5** (250 and 62.5 MHz, respectively, recorded in CD_2Cl_2)

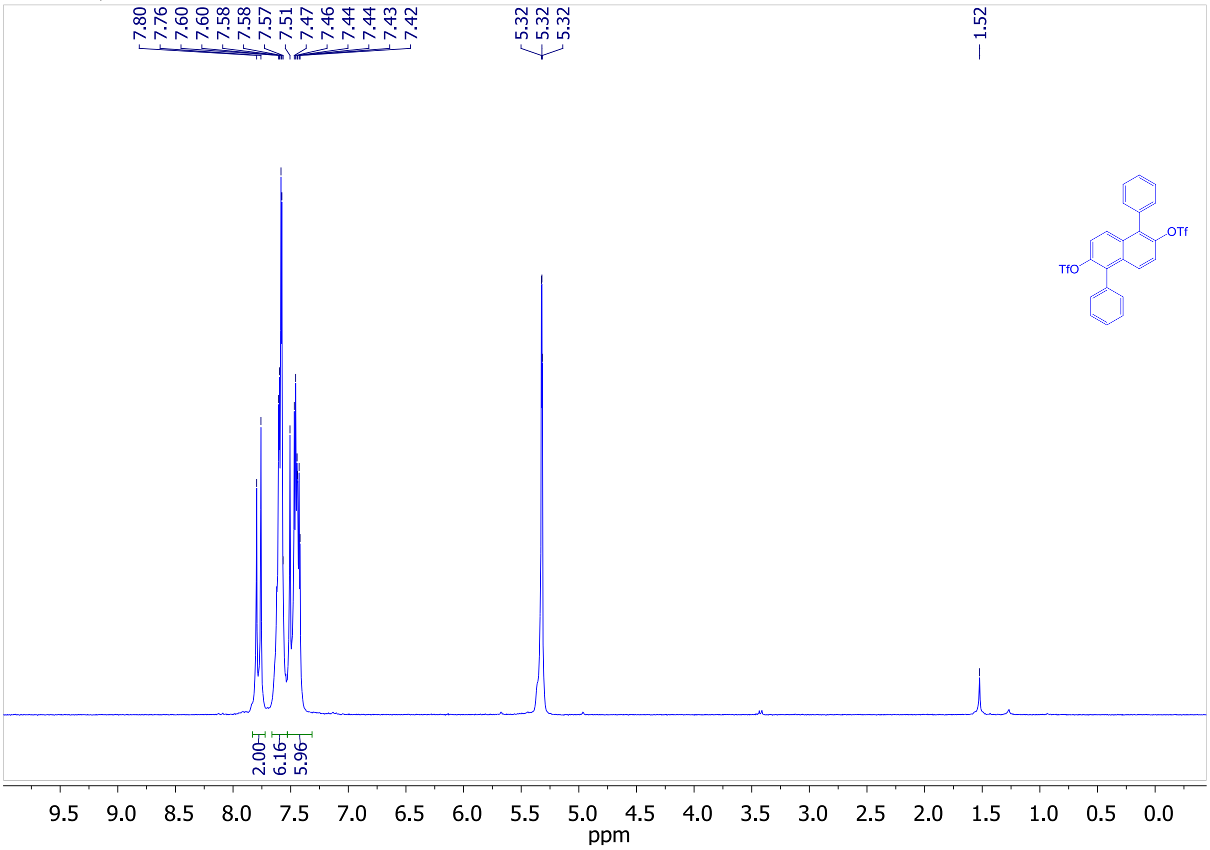

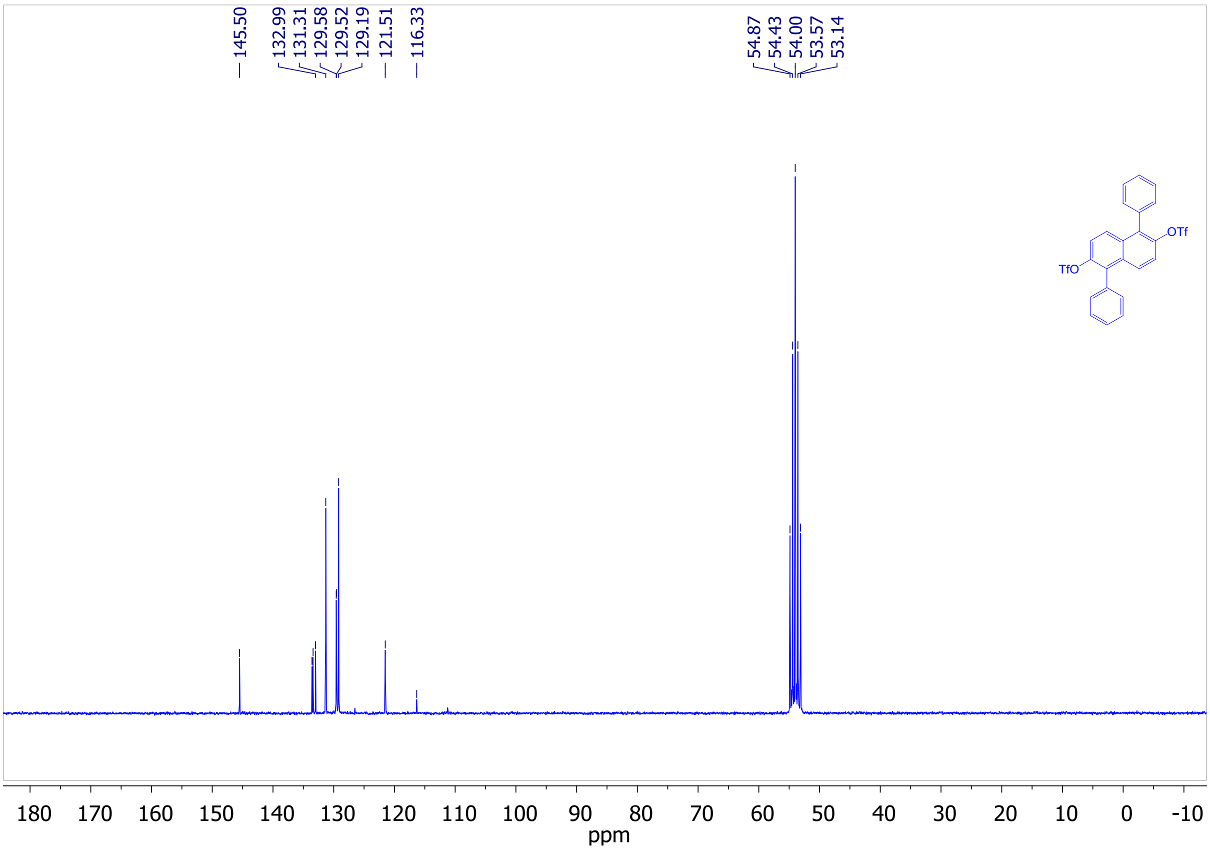

5.6. ^{1}H and ^{13}C NMR spectra of compound **6** (250 and 62.5 MHz, respectively, recorded in CD_2Cl_2)

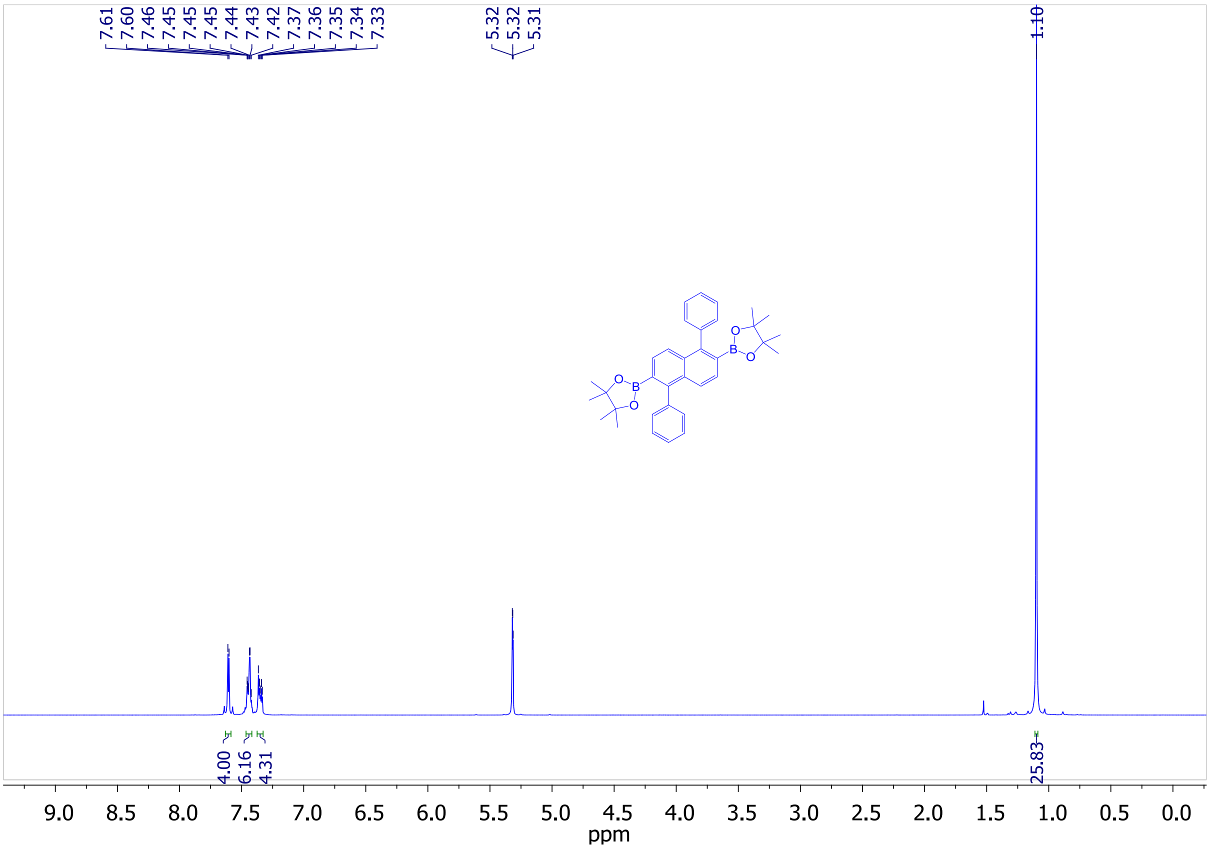

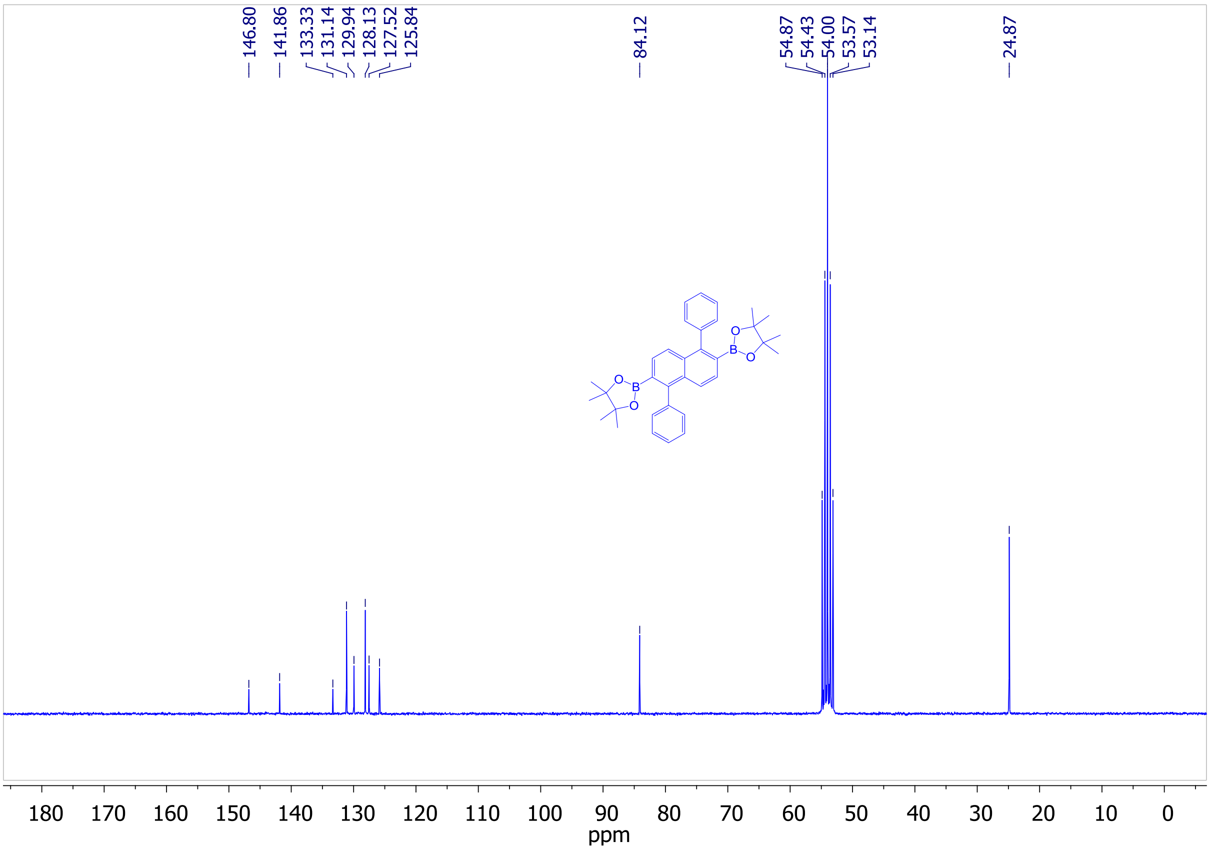

5.7. ^{1}H and ^{13}C NMR spectra of compound **7** (250 and 62.5 MHz, respectively, recorded in CD_2Cl_2)

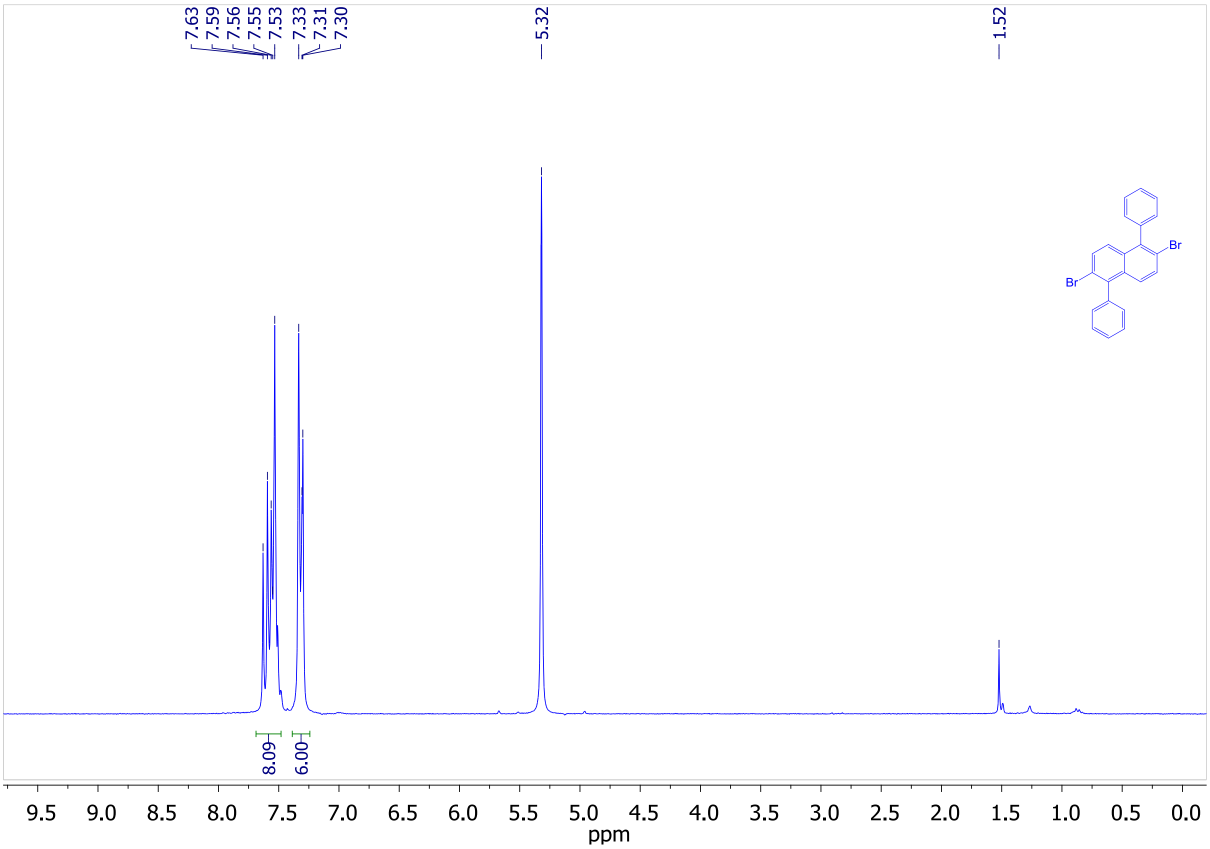

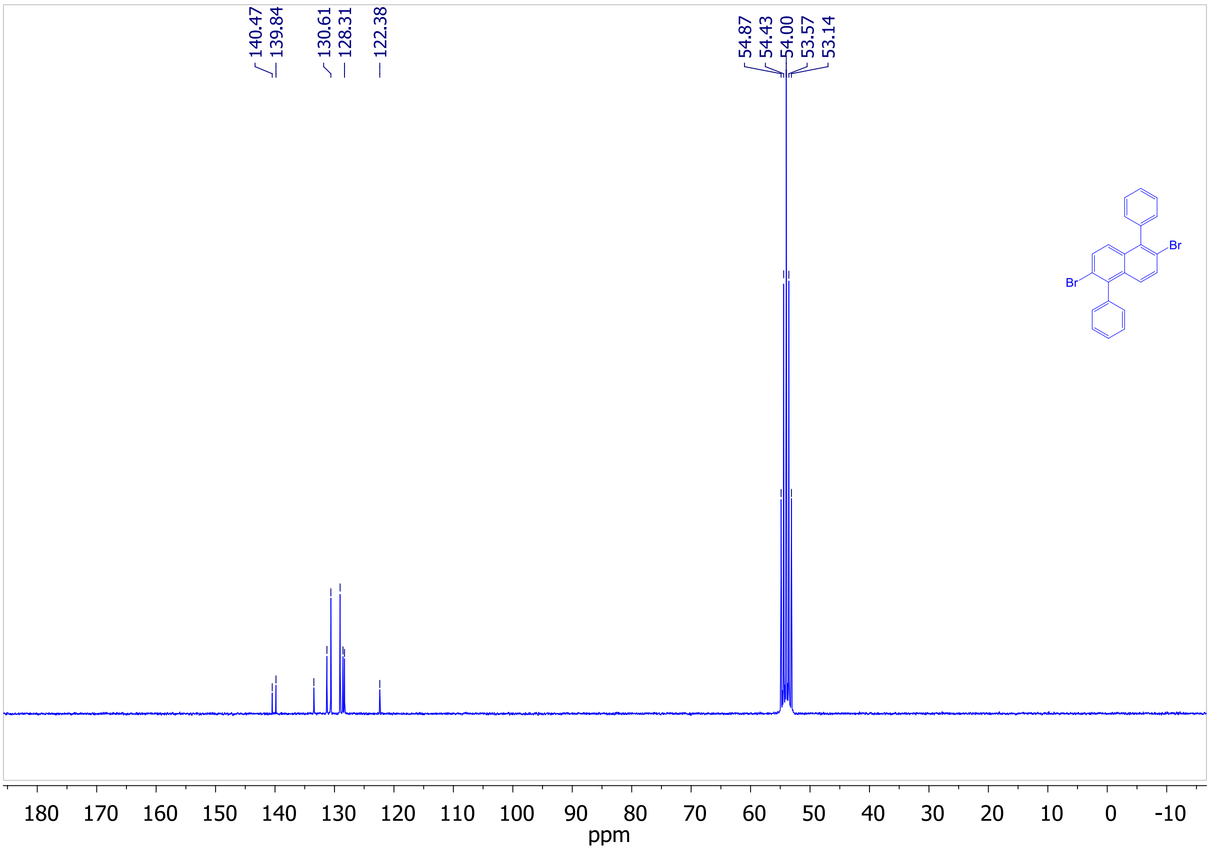

6. References:

[1] J.-Y. Wang, Y. Zhou, J. Yan, L. Ding, Y. Ma, Y. Cao, J. Wang and J. Pei, *Chemistry of Materials* **2009**, *21*, 2595-2597.
